# A knockin mouse model for human *ATP4a^R703C^* mutation identified in familial gastric neuroendocrine tumors recapitulates the premalignant condition of the human disease and suggests new therapeutic strategies

**DOI:** 10.1242/dmm.025890

**Published:** 2016-09-01

**Authors:** Oriol Calvete, Andrea Varro, D. Mark Pritchard, Alicia Barroso, Marta Oteo, Miguel Ángel Morcillo, Pierfrancesco Vargiu, Steven Dodd, Miriam Garcia, José Reyes, Sagrario Ortega, Javier Benitez

**Affiliations:** 1 Human Genetics Group, Spanish National Cancer Research Center (CNIO), Madrid 28029, Spain; 2 Spanish Center for Biomedical Network Research on Rare Diseases (CIBERER), Madrid 28029, Spain; 3 Department of Cellular and Molecular Physiology, Institute of Translational Medicine, University of Liverpool, Liverpool L69 3BX, UK; 4 Centro de Investigaciones Energéticas, Medioambientales y Tecnológicas (CIEMAT), Madrid 28040, Spain; 5 Transgenic Mice Core Unit, Spanish National Cancer Research Center (CNIO), Madrid 28029, Spain; 6 Animal Facility Core Unit, Spanish National Cancer Research Center (CNIO), Madrid 28029, Spain; 7 Department of Gastroenterology, Hospital INCA, Majorca 07300, Spain

**Keywords:** *ATP4a*, Gastric carcinoids, Achlorhydria, Hypergastrinemia, Oxyntic glands

## Abstract

By whole exome sequencing, we recently identified a missense mutation (p.R703C) in the human *ATP4a* gene, which encodes the proton pump responsible for gastric acidification. This mutation causes an aggressive familial type I gastric neuroendocrine tumor in homozygous individuals. Affected individuals show an early onset of the disease, characterized by gastric hypoacidity, hypergastrinemia, iron-deficiency anemia, gastric intestinal metaplasia and, in one case, an associated gastric adenocarcinoma. Total gastrectomy was performed as the definitive treatment in all affected individuals. We now describe the generation and characterization of a knockin mouse model for the *ATP4a^R703C^* mutation to better understand the tumorigenesis process. Homozygous mice recapitulated most of the phenotypical alterations that were observed in human individuals, strongly suggesting that this mutation is the primary alteration responsible for disease development. Homozygous mice developed premalignant condition with severe hyperplasia, dysplasia and glandular metaplasia in the stomach. Interestingly, gastric acidification in homozygous mice, induced by treatment with 3% HCl acid in the drinking water, prevented (if treated from birth) or partially reverted (if treated during adulthood) the development of glandular metaplasia and dysplasia in the stomach and partially rescued the abnormal biochemical parameters. We therefore suggest that, in this model, achlorhydria contributes to tumorigenesis to a greater extent than hypergastrinemia. Furthermore, our mouse model represents a unique and novel tool for studying the pathologies associated with disturbances in gastric acid secretion.

## Abstract (precis)

**Summary:** Gastric pathologies in an *ATP4a* knockin mouse model of a mutation responsible for the development of gastric neuroendocrine tumors in humans are prevented and reverted by adding HCl to drinking water.

## INTRODUCTION

Type I gastric neuroendocrine tumors (gNETs, also known as gastric carcinoids) are multifocal cellular lesions, usually benign in behavior and associated with hypochlorhydria and hypergastrinemia (Zhang et al., 2011; Vannella et al., 2012). In normal physiological conditions, gastrin is secreted by the G cells located in the antrum of the stomach (pyloric glands), and stimulates acid secretion by the gastric oxyntic glands, which are located in the corpus region of the stomach. These glands are mainly composed of chief (or zymogenic) cells, enterochromaffin-like cells (ECL cells) and parietal cells (PCs). Gastric acid secretion is achieved through proton pumps (encoded by the *ATP4a* gene) located in PCs. Gastric acid is also needed for the conversion of pepsinogen to pepsin (activated form), and facilitates iron absorption in the duodenum (Schubert, 2015). In addition, PCs in humans produce intrinsic factor (IF), which is necessary for the absorption of vitamin B12.

Chronic hypergastrinemia associated with chronic atrophic gastritis, either of autoimmune origin or as a result of *Helicobacter pylori* infection, results in ECL cell hyperplasia, which is a precursor of gastric NET development in some individuals. Sporadic type I gastric NETs are associated with progressive destruction of PCs and therefore with gastric hypoacidity and megaloblastic anemia caused by vitamin B12 malabsorption.

The management of type I gastric NETs is still open to debate because of their usual relatively benign clinical course (Gladdy et al., 2009). Endoscopic mucosal resection is often performed (Uygun et al., 2014); however, the recurrence rate is high (up to 68%) (Merola et al., 2012). Surgical approaches such as antrectomy to remove the source of hypergastrinemia are required for larger polyps or for aggressive forms of the tumors showing mucosal invasion (Nikou and Angelopoulos, 2012).

Gastrin/CCK-2 receptor antagonist drugs such as netazepide (Fossmark et al., 2012; Moore et al., 2013) or antibodies against progastrin-releasing peptides have also been shown to inhibit ECL cell hyperplasia. Somatostatin analogs, which inhibit gastrin production, have also been proposed as treatment, in view of their antisecretory, antiproliferative and antiangiogenic effects (Culler et al., 2011). However, these drugs require long-term treatment and are also very expensive (Massironi et al., 2015).

By whole exome sequencing (WES), we recently found a missense mutation in the *ATP4a* gene (p.R703C), which encodes the proton pump in PCs. This mutation was responsible for the development of an aggressive form of type I gastric NET in homozygous individuals within a consanguineous family (Calvete et al., 2015). This atypical gastric NET had a very poor prognosis. The five affected family members required total gastrectomy, had nodal infiltration and one individual also developed gastric adenocarcinoma. Additionally, the disease was associated with an earlier age of onset (relative to other types of gNETs) and with iron-deficiency anemia rather than megaloblastic anemia. Different to the classic type I gastric NETs, PCs were still observed in the non-tumor gastric corpus mucosa and IF was produced normally. However, these individuals did not produce gastric acid normally and this resulted in hypergastrinemia. The clinical features of these individuals have been described in detail in Calvete et al. (2015). The only treatment currently available for these aggressive gastric NETs involves total gastrectomy. We have now generated a knockin mouse model carrying the equivalent mutation to the one found in these individuals in order to investigate its causal effects upon the development of the disease and for testing potential alternative therapeutic strategies.

## RESULTS

### Phenotype of the ATP4a p.R702C knockin mice

The recessive mutation found in the *ATP4a* gene (p.R703C) responsible for atypical type I familial gastric NETs (Calvete et al., 2015) corresponds to the p.R702C mutation in mice. We converted the wild-type (WT) CGA (Arg) codon to TGC (Cys) to generate the mutated *Atp4a* knockin (KI) allele (Fig. S1) by homologous recombination in embryonic stem cells (ESC) (see Materials and methods). Homozygous animals for the knockin allele (KI/KI) did not show obvious developmental defects and were fertile. They also had normal behavior, weight and feeding habits.

For most assays, three mice of each gender were tested at each selected time point. Unless otherwise indicated, data from males and females were analyzed together to improve statistical power (*n*=6). The phenotype was already evident at 80 days of life and did not significantly change over time. We consider the last measured time point (>350 days) as being the most representative of the pathology development (Table 1).

**Table 1.**
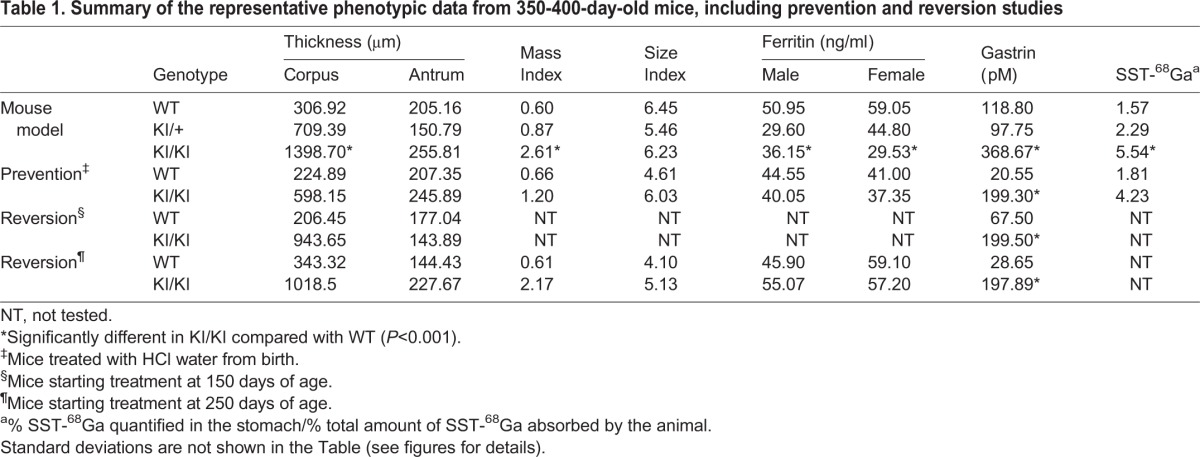
**Summary of the representative phenotypic data from 350-400-day-old mice, including prevention and reversion studies**

**Fig. 1.**
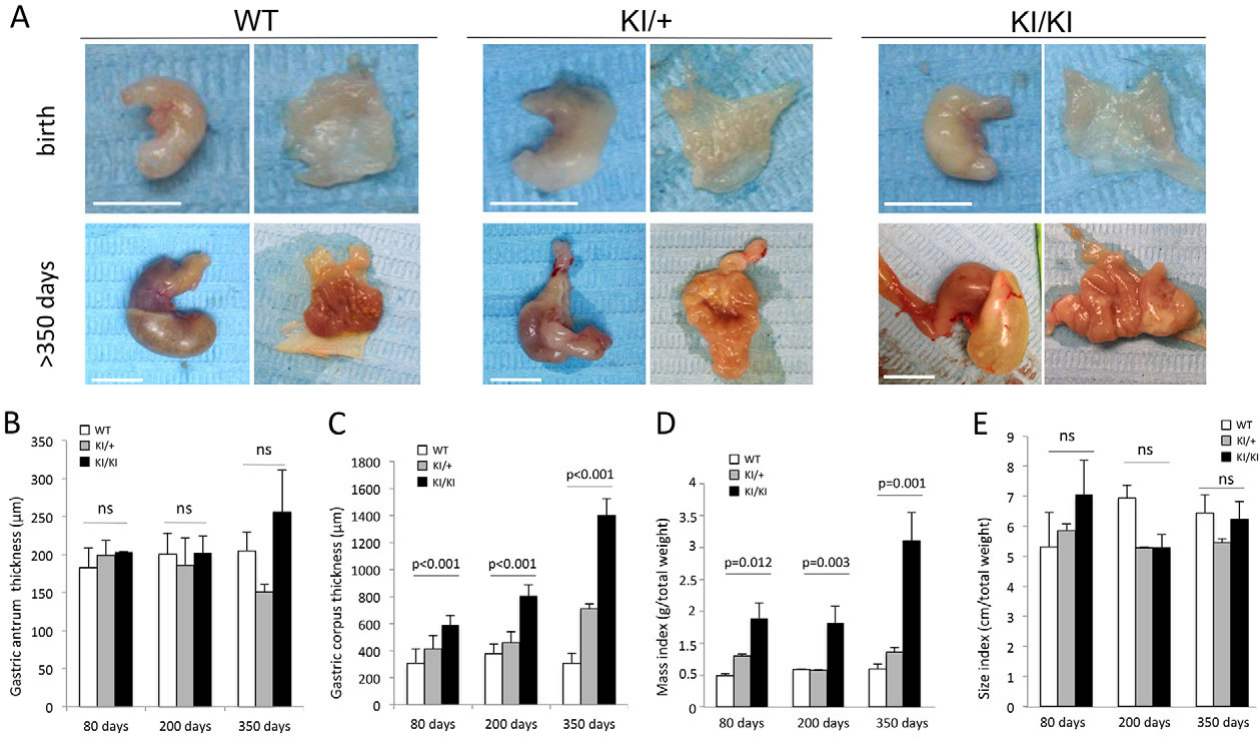
**Stomach hyperplasia in mutant mice.** (A) Macroscopic external (left) and internal (right) images of WT, KI/+ and KI/KI stomachs. Images are representative examples of stomachs from mice at birth (top) and more than 1 year old (bottom). Scale bar: 1 cm. (B) Gastric antrum thickness (μm) over time. (C) Gastric corpus thickness (μm) over time. (D) Stomach mass index (weight of the stomach normalized by the total weight of the animal) over time. (E) Size index (size of the stomach normalized by the total weight of the animal) over time. *n*=6 per genotype. ns, not significant. Data in B-E are represented as mean±s.d.

Homozygous (KI/KI) mice for the *Atp4a* p.R702C mutation showed a pronounced overall thickness and weight of their stomachs (Fig. 1A). Only a slight increase in antrum thickness was observed in the KI/KI mice, however, the corpus thickness were severely increased (*P*<0.001) compared with WT mice (Fig. 1B,C). No significant differences were observed between WT and heterozygous (KI/+) stomachs. In addition, no differences were found in the non-glandular regions of the stomach (close to the pylorus and duodenum) among the three genotypes (Table 1).

When both weight and size of the stomachs were normalized to the total weight of the animals (values called mass and size index, respectively), mass index was significantly increased (*P*<0.012) in KI/KI compared with WT mice (Fig. 1D). However, no differences were observed in size index (Fig. 1E) suggesting that there is a thickening of the stomach wall towards the lumen without altering the total volume of the organ.

### Histopathology of the gastric wall in homozygous mutant mice

**Fig. 2.**
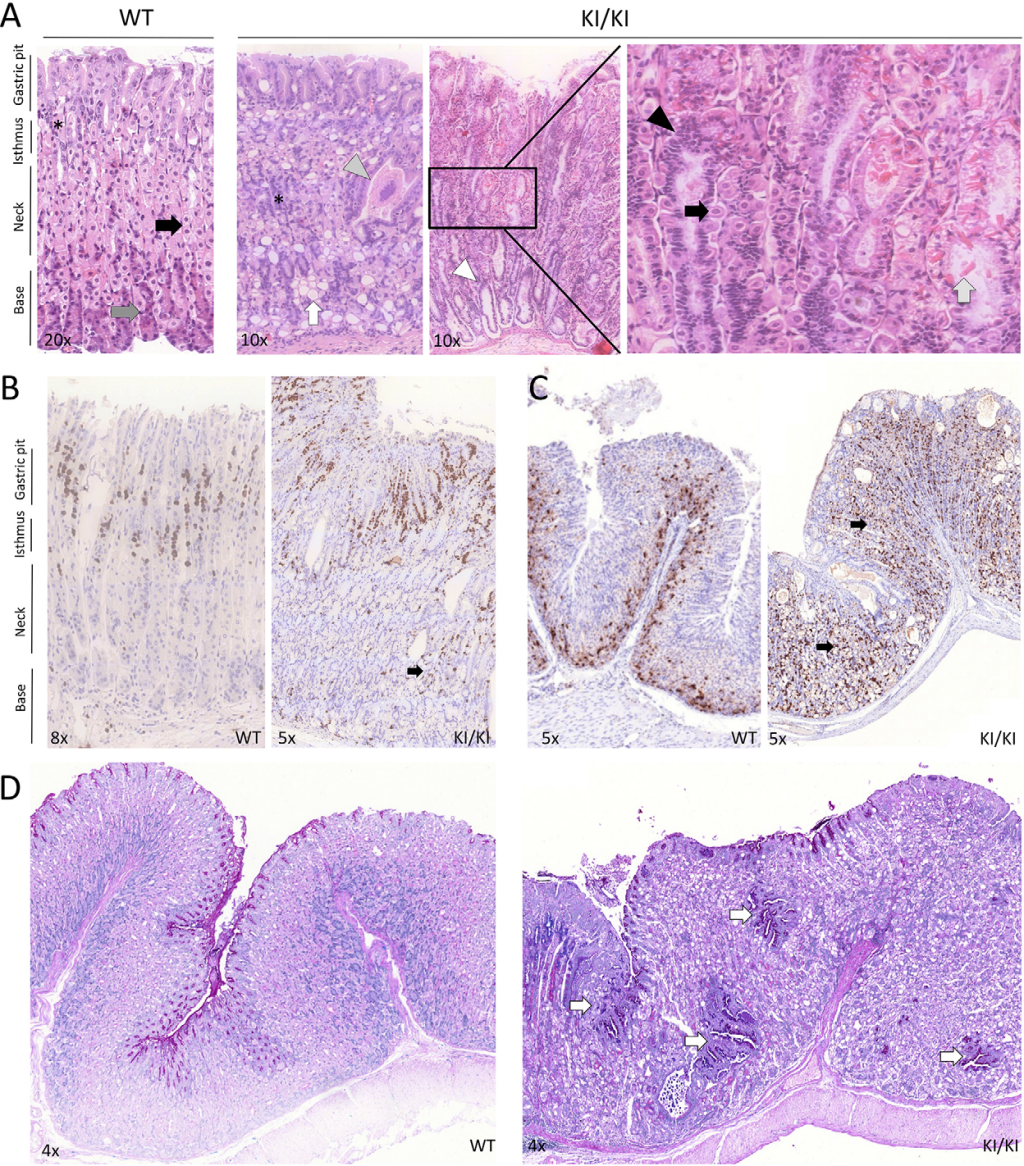
**Histological alterations in the corpus region of the stomach.** (A) Representative H&E staining of a transverse section of stomach from mice at one year of age. In WT mice, progenitor cells are located in the isthmus (asterisk), PCs (basic staining) stain magenta with H&E and are located in the neck (black arrow). ECL cells and zymogenic cells (acid staining) stain dark purple with H&E and are located at the base of oxyntic glands (gray arrow). In KI/KI mice, cellular disorganization and increased thickness of the stomach wall were observed, characterized by the presence of proliferating cells (at isthmus in WT mice) and ECL-cells (at base in WT mice) within the neck region of gastric corpus glands (asterisk). In addition, high numbers of epidermoid cysts surrounded by a single layer of cuboidal epithelium (gray arrowhead), vacuolization of gastric mucosal cells (white arrow) and widely opened lumen of the oxyntic glands (white arrowhead) were seen in KI/KI mice. In the enlarged image at the far right, increased number of grouped cells from the isthmus region (black arrowhead), dysplastic cytoplasms of PCs (black arrow) and hyalinosis (light gray arrow), indicating a premalignant condition, are shown. (B) IHC of gastric corpus stained with anti-Ki67 antibody. In WT mice, the staining is limited to the isthmus region, which is the proliferative zone. Increased Ki67 staining is observed in the KI/KI stomach isthmus, but is also seen in the neck region (black arrow). (C) IHC of gastric corpus stained with anti-chromogranin A antibody. In WT mice, the staining is limited to the base of the oxyntic glands whereas in KI/KI stomachs it is also seen in the entire neck region (black arrows). (D) Alcian Blue-PAS staining of the corpus region of the stomach of WT (left) and KI/KI (right) mice. White arrows show areas of glandular metaplasia in the KI/KI stomach.

The gastric wall hyperplasia observed in KI/KI mice was evaluated by hematoxylin and eosin (H&E) staining. In WT stomachs, PCs stained eosinophilic with H&E and were found in the neck region of the oxyntic glands (Fig. 2A). Chief (zymogenic) cells stained basophilic with H&E and were located at the gland base (Fig. 2A).

In contrast, the KI/KI stomachs showed glandular disorganization and the integrity and architecture of the oxyntic glands were compromised. A higher cellularity, loss of nuclear polarity, increased number of inflammatory cells, swollen cytoplasms and loss of normal gland morphology were observed in the gastric corpus of KI/KI mice. Basophilic staining with H&E was no longer limited to the base of the oxyntic glands, but instead extended to the neck region (Fig. 2A). Cysts and vacuolization of gastric mucosa cells were also observed.

The mislocalized cells in the KI/KI stomachs were further analyzed by immunohistochemistry (IHC). The hybridization signal with anti-Ki67 (a proliferation marker) in the gastric corpus of KI/KI mice was not only observed in the isthmus region (the normal proliferative zone in WT mice) but also extended along the whole neck and base of the oxyntic glands (Fig. 2B). ECL cells (chromogranin A-positive staining, Table S1) were localized at the base of the gastric oxyntic glands in WT stomachs (Fig. 2C). However, in KI/KI mice, the staining pattern was no longer limited to the base, but was also extended through the neck region of the mucosa. Moreover, there were an increased number of chromogranin A-positive cells suggesting that ECL cell number contributes towards the total mucosa hyperplasia observed in KI/KI mice.

In humans who are homozygous for the mutation, the gastric somatostatin receptor (SSTR2) was found to be overexpressed in gastric ECL cells (Calvete et al., 2015). In contrast, in KI/KI mice, no SSTR2 overexpression was observed by IHC (data not shown). The SSTR2 signal was also evaluated by PET-CT imaging. A SST analogue labeled with gallium-68 (SST-^68^Ga) was used to detect differences in the expression of this receptor in KI/KI mice. No differences in the amount of SST-^68^Ga/gram of stomach were observed between WT (Movie 1) and KI/KI animals (Movie 2). However, the SST-^68^Ga signal in the stomach was increased in KI/KI mice when normalized for the total amount of SST-^68^Ga absorbed by the animal (Table 1). This observation, together with the increased number of chromogranin A-positive cells, suggests that there is an increase in the number of cells expressing this receptor rather than an increased number of receptors per cell as observed in humans.

The gastric mucosa was also evaluated with Alcian Blue-PAS staining. Blue staining, which localized to the base of the gastric mucosa in WT stomachs, was not observed in KI/KI stomachs, which showed more irregular structure of the glands (Fig. 2D). Moreover, mucous metaplasia was found in KI/KI mice.

In summary, we observed loss of architectural integrity of the oxyntic glands in KI/KI stomachs that showed several similarities to the histopathological changes previously reported in the non-neuroendocrine tumor-affected areas of the gastric mucosa in individuals harboring the same mutation (Calvete et al., 2015).

### Gastric acid, serum gastrin concentration and anemia

The individuals described in Calvete et al. (2015) showed gastric achlorhydria and developed iron-deficiency anemia and hypergastrinemia. We therefore proceed to study these parameters in mutant mice.

**Fig. 3.**
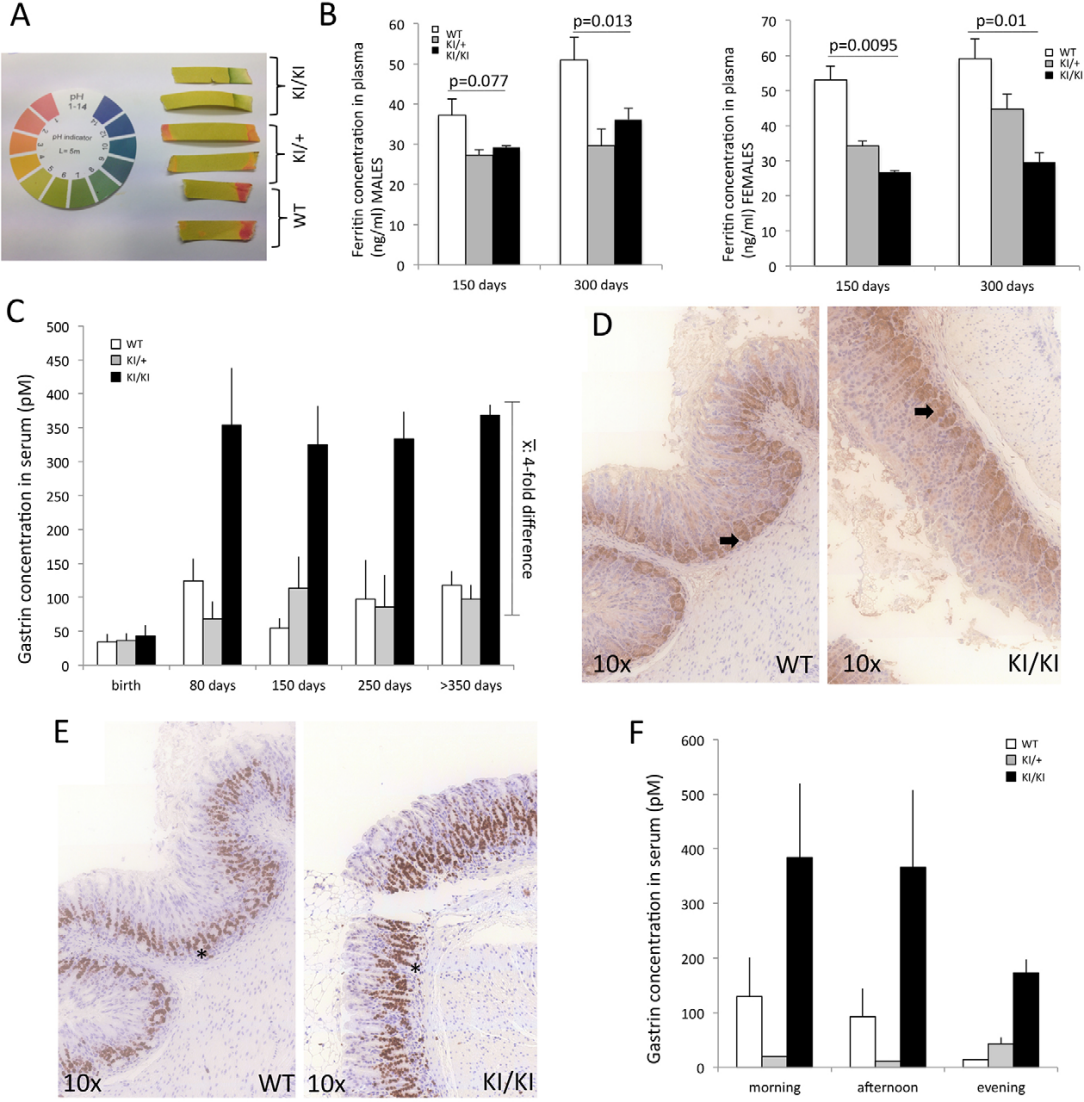
**Hypoacidity, anemia and hypergastrinemia in KI/KI mice.** (A) Gastric pH values determined by direct measurement with colorimetric paper at necropsy. Measurements of two different animals are shown for each genotype; gastric pH values were ∼1, ∼4 and ∼7 for WT, KI/+ and KI/KI mice, respectively. (B) Ferritin concentrations in plasma (mM) of WT, KI/+ and KI/KI male (left) and female (right) mice. (C) Gastrin concentrations in serum (pM) of WT, KI/+ and KI/KI mice at ages indicated. (D) Representative IHC images of the antrum region of the stomach stained with anti-gastrin antibody in WT and KI/KI mice at 350 days of age. In both genotypes, the anti-gastrin antibody labels G-cells at the base of pyloric glands (black arrows). (E) IHC with anti-Ki67 antibody of the gastric antrum from representative WT and KI/KI mice. In both genotypes, progenitor cells are located at the base of pyloric glands (asterisks). (F) Gastrin concentrations in serum (pM) of WT, KI/+ and KI/KI mice. *n*=6 per genotype. ns, not significant. Data in B,C,F are represented as mean±s.d.

Gastric pH was directly measured from the stomach content of mice at necropsy. No differences in pH were observed related to the age at the time of euthanasia and/or to the gender of the animals for any of the genotypes. However, highly increased gastric pH was observed in all KI/+ (pH 4) and KI/KI (pH 7) animals compared with WT mice (pH 1.5-2.5; Fig. 3A).

Anemia in the mice was evaluated by measuring ferritin concentration (ng/ml) in plasma. This parameter was observed to be different between genders (as expected) and was, therefore, analyzed separately in male and female mice. Significant differences in plasma ferritin concentration were found between sex-matched KI/KI animals compared with WT. This difference was more pronounced in females (*P*<0.01 for all measurements) than in males (*P*=0.013 at 300 days; Fig. 3B, Table 1).

No difference in serum gastrin concentration was observed among the different genotypes at birth; however, marked hypergastrinemia (fourfold increase in mean serum gastrin concentration) was observed in KI/KI animals, relative to KI/+ and WT mice, at all the other time points analyzed (Fig. 3C, Table 1). IHC of the antrum region of the stomach, where the G-cells are located, using both anti-gastrin and anti-Ki67 antibodies (Table S1), showed no overexpression of gastrin or increased proliferation of G-cells in KI/KI compared with WT mice (Fig. 3D and E, respectively).

WT animals showed the normal variation in serum gastrin concentration that is associated with feeding, with reduced concentrations being observed during the non-feeding period (daytime). However, high serum gastrin concentrations persisted in KI/KI animals throughout the day and were not affected by feeding to the same extent as WT animals (Fig. 3F). This suggests that there is an increased secretion of gastrin throughout the day in KI/KI animals, secondary to hypochlorydria and not just in response to feeding.

In summary, KI/KI mice mimic all biochemical features of human individuals homozygous for the *ATP4a* gene mutation (achlorhydria, hypergastrinemia and iron-deficiency anemia). This strongly suggests that the tested mutation in the *ATP4a* gene is primarily responsible for the development of these alterations.

### Prevention of the phenotypic changes in KI/KI mice

**Fig. 4.**
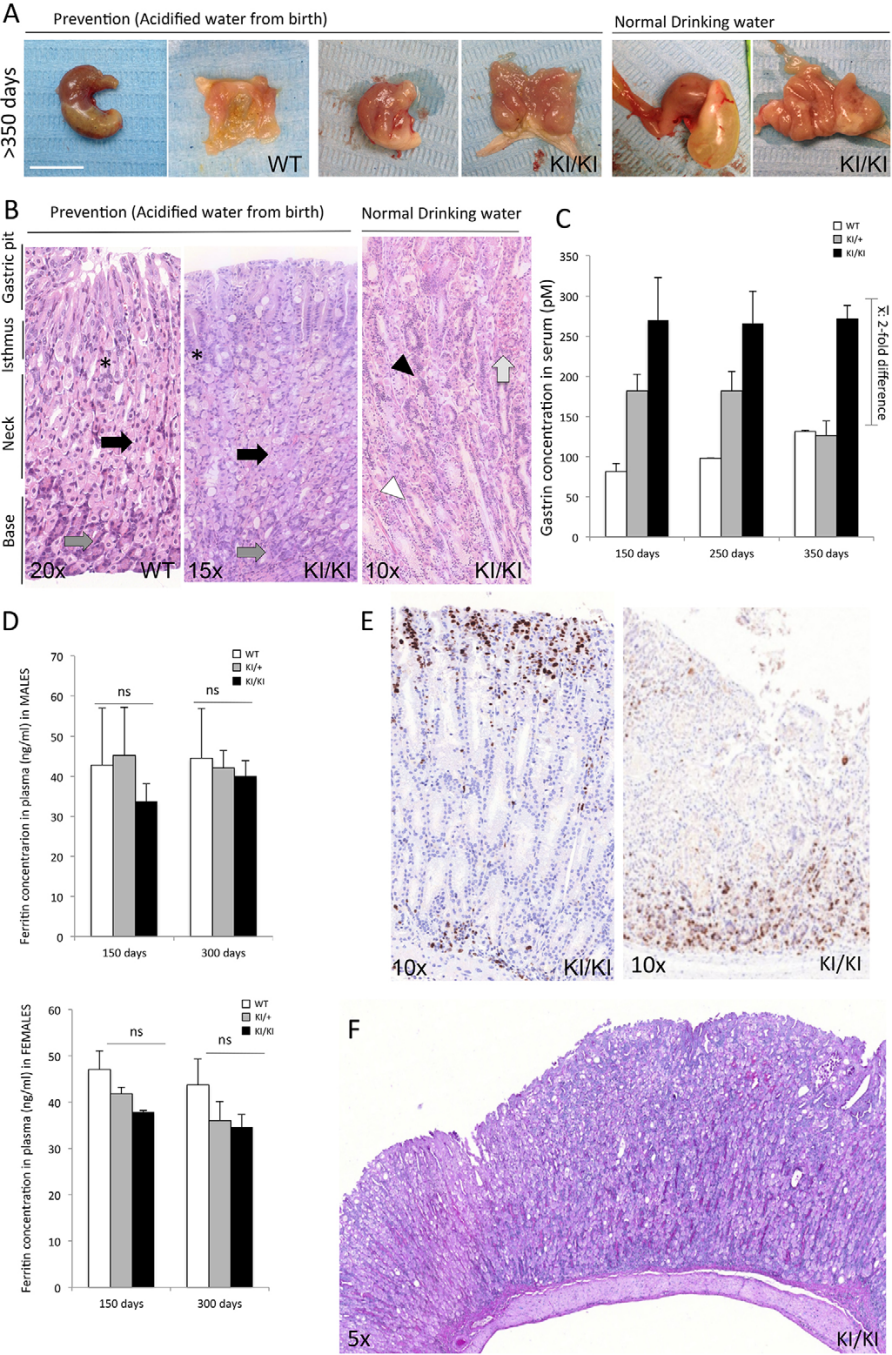
**Phenotype prevention by treatment with HCl water.** (A) Representative macroscopic external (left) and internal (right) images of stomachs are shown for HCl water­-treated and untreated WT and KI/KI mice at >350 days of age. No lesions or injuries were observed in WT mice following this treatment. Gastric hyperplasia in treated KI/KI mice (pH 4) is drastically reduced compared with KI/KI mice without treatment (pH 7). Scale bar: 1 cm. (B) Representative H&E staining images of KI/KI stomachs from treated mice compared with WT and non-treated KI/KI mice at 350 days of age. Similar architecture consisting of progenitor neck mucosa cells located in the isthmus (asterisks), PCs in the neck (black arrows), and ECL and zymogenic cells in the base of oxyntic glands (dark gray arrows) were observed in WT and treated KI/KI stomachs. Widely opened oxyntic glands (white arrowhead), grouped nuclei from the isthmus region (black arrowhead) and hyalinosis (light gray arrow), observed in untreated KI/KI stomachs, were not detected in treated mice. (C) Serum gastrin concentrations (pM) in WT, KI/+ and KI/KI mice treated for the times indicated. (D) Ferritin concentrations in plasma (µM) of treated WT, KI/+ and KI/KI males (top) and females (borrom). (E) IHC of corpus region of a representative treated KI/KI stomach with anti-Ki67 (left) and anti-chromogranin A (right) antibodies. Staining is limited to the isthmus and the base of the oxyntic glands, respectively, as observed in WT mice (Fig. 2B). (F) Alcian Blue-PAS staining of the corpus region of a representative KI/KI stomach from a 350-day-old mouse. *n*=6 per genotype. ns, not significant. Data in C,D are represented as mean±s.d.

In order to analyze the impact of low stomach pH on the development of the observed phenotypes in these mice, HCl-acidified drinking water (pH 2.6-2.8) was initially given to the animals from birth. The gastric pH of all mice treated with acidified water was 2.5 and 4 for KI/+ and KI/KI animals, respectively. The treatment with acidified water did not cause macroscopic lesions in the stomach (see treated WT stomach in Fig. 4A) or esophagus (Fig. S2).

Gastric hyperplasia was markedly reduced in acidified water-treated KI/KI mice compared with untreated KI/KI mice. Ultrasound echography also confirmed that gastric corpus thickness was reduced in treated compared with untreated KI/KI mice. The mass index was also partially restored (Table 1). H&E-stained stomachs of these treated mice were also the subject of histological examination. A slight gastric hyperplasia and some cells with dilated cytoplasms were still observed. However, no inflammation, loss of cellular polarity or any kind of metaplasia were found in the stomachs of treated KI/KI mice. Moreover, the normal oxyntic gland architecture was restored (Fig. 4B).

Likewise, treatment of KI/KI mice with acidified water also led to normalization of biochemical parameters. The extent of hypergastrinemia was reduced in acidified water-treated KI/KI mice (Fig. 4C, Table 1). Gastrin concentration was only twofold higher in treated KI/KI mice than in WT, instead of the fourfold difference observed for non-treated mice (Fig. 3C). No significant differences were found in ferritin concentrations between KI/KI and WT mice, of either gender, following acidified water treatment (Fig. 4D). Thus, administration of acidified water was sufficient for the restoration of iron absorption and prevented the onset of iron-deficiency anemia in KI/KI mice.

The mislocalized hybridization signals observed in untreated KI/KI mice (described above) were also partially normalized following treatment with acidified water. Anti-Ki67 and anti-chromogranin A immunostaining were mainly observed at the isthmus and the base of the gastric mucosa, respectively, in treated KI/KI mice (Fig. 4E), as it was observed in WT mice. The gastric mucous metaplasia that was observed by Alcian Blue-PAS-staining in KI/KI mice was no longer observed either in acidified water-treated mice (Fig. 4F).

Finally, the ratio of SST-^68^Ga in the stomach to total SST-^68^Ga absorbed was reduced in treated KI/+ and KI/KI mice compared with non-treated mice (Table 1), again suggesting that the prevention of the histological alterations and normalization of the cell population was achieved in mutant mice by stomach pH acidification.

### Reversion of phenotypic changes in KI/KI mice

**Fig. 5.**
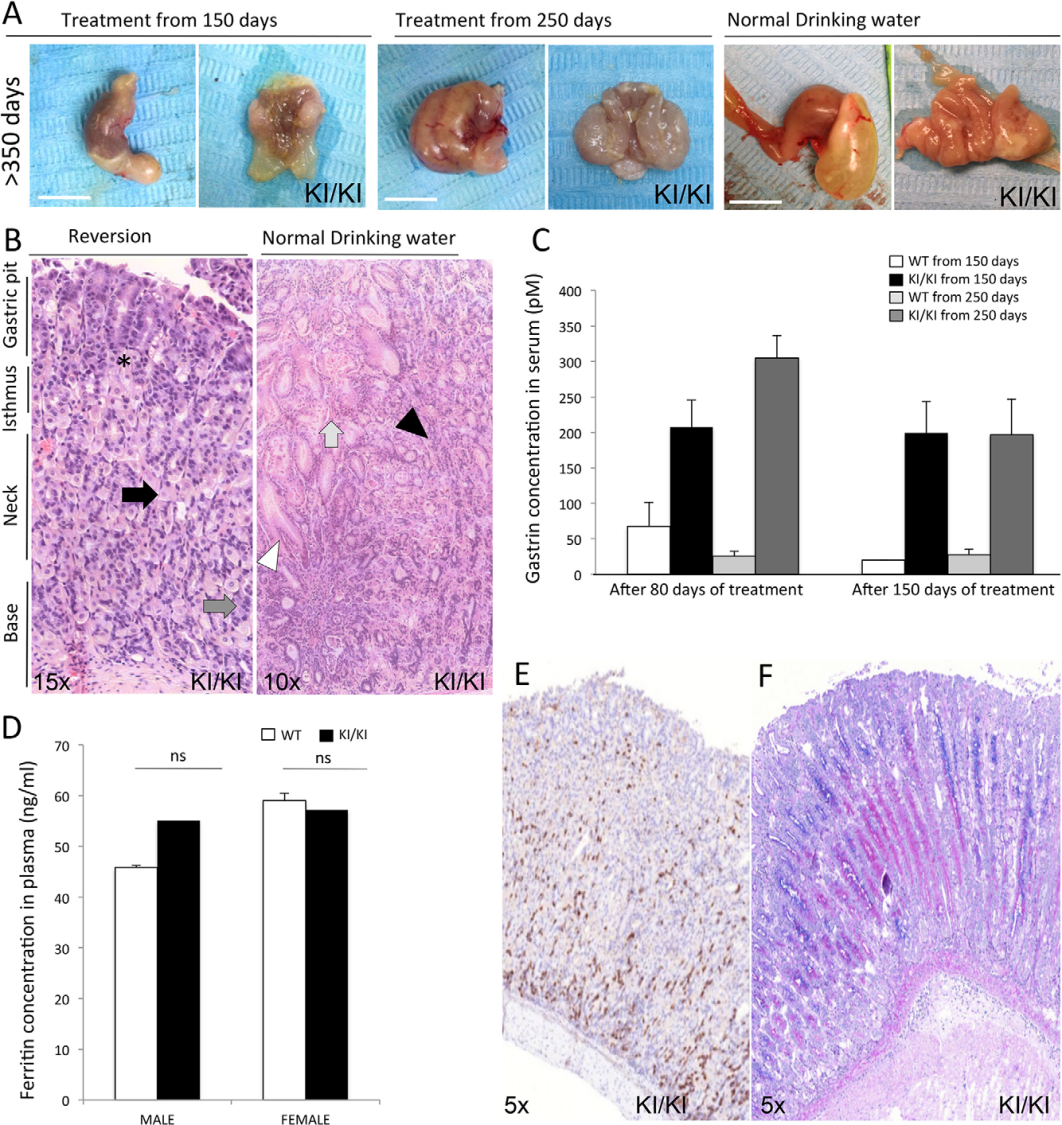
**Phenotype reversion by treatment with HCl water.** (A) Representative macroscopic external (left) and internal (right) images of stomachs are shown for KI/KI mice after 150 days of treatment starting at 150 or 250 days of age. Gastric hyperplasia in KI/KI mice treated (pH 4) from day 150 was drastically reduced compared with untreated KI/KI mice (pH 7). The extent of gastric hyperplasia reduction was less evident in mice that started treatment at 250 days of age. See Table 1 for gastric thickness data. Scale bar: 1 cm. (B) Corpus region of a stomach from a KI/KI mouse treated for 150 days starting at 150 days of age stained with H&E. Cellular organization was partially restored in treated mice. Major lesions also disappeared in these mice compared with KI/KI mice without treatment. In treated KI/KI stomachs, progenitor neck mucosa cells were located in the isthmus (black asterisk), PCs in the neck (black arrow), and ECL and zymogenic cells at the base of oxyntic glands (dark gray arrow). Widely opened oxyntic glands (white arrowhead), grouped nuclei from the isthmus region (black arrowhead) and hyalinosis (light gray arrow), observed in KI/KI stomachs, were not observed in treated KI/KI stomachs. (C) Serum gastrin concentrations (pM) in WT and KI/KI mice treated for 80 and 150 days of treatment. (D) Ferritin concentrations in plasma (µM) for male and female mice after 150 days of treatment. (E) IHC staining with anti-chromogranin A antibody of the corpus region of a representative KI/KI stomach after 150 days of treatment. (F) Alcian Blue-PAS staining of the corpus region of a representative KI/KI stomach after 150 days of treatment. *n*=6 per genotype. ns, not significant. Data in C,D are represented as mean±s.d.

In order to explore the potential of the stomach acidification treatment as a therapeutic approach, we next wanted to evaluate whether treatment with acidified water could not only prevent, but also revert the development of the malignant phenotype of homozygous mutant mice. For this purpose, treatment with acidified drinking water (pH 2.6-2.8) was started when mice were 150 or 250 days old and the effects were evaluated after 80 and 150 days of treatment in both cases. Gastric pH was partially restored in all treated mice; pH values of 2.5 and 4 were obtained for all KI/+ and KI/KI treated animals, respectively. After 150 days of treatment, starting both at 150 and 250 days of life, abnormal gastric morphology of the stomach was still found; however, the extent of gastric hyperplasia was reduced in treated versus non-treated KI/KI mice (Fig. 5A). Larger responses were observed in the mice that started treatment at 150 days of age compared with those that started at 250 days of age. As in the prevention studies, gastric thickness and mass index were also partially restored compared with non-treated KI/KI mice (Table 1).

Recovery of gastric histopathological changes was also observed in KI/KI mice after 150 days of treatment, although a certain degree of gastric disorganization and vacuolization still remained (Fig. 5B). Additionally, gastrin and ferritin concentrations were partially restored in treated versus non-treated KI/KI mice (Fig. 5C,D, Table 1). Serum gastrin concentrations decreased to a lesser extent in mice that started treatment at 250 days of age compared with those that started treatment at 150 days. However, in both scenarios, reduced serum gastrin concentrations were observed after 150 days of treatment (Fig. 5C), similar to the levels observed in the prevention study.

Immunohistochemistry using the anti-chromogranin A antibody also showed a partially normalized appearance in treated KI/KI mice (Fig. 5E). Most of the staining was observed at the base of the oxyntic glands as seen in WT mice. However, a certain degree of mislocalization through the gastric neck region still remained. Metaplasia was no longer observed by Alcian Blue-PAS staining in the gastric mucosa of treated KI/KI mice (Fig. 5F). All these data indicate that the phenotype of KI/KI mice can also be reverted, at least to some extent, by normalization of the gastric pH and thus suggest gastric acidification as a potential alternative for therapeutic treatment of patients carrying this mutation.

## DISCUSSION

A knockin mouse line carrying the p.R702C mutation in the *Atp4a* gene in homozygosis, although normal at birth, developed most of the clinical features and the premalignant condition previously described in human individuals who are homozygous for the same mutation (Calvete et al., 2015). These include achlorhydria, hypergastrinemia, iron-deficiency anemia and gastric histopathological lesions. The stomachs of adult KI/KI mice exhibited severe hyperplasia, metaplasia and oxyntic glandular structural disorganization with dysplastic gastric glands. After one year, the mice did not develop gastric NETs; however, premalignant pathology and cellular dysplasia, similar to those observed in the non-tumor affected areas of the stomachs of human individuals, were found. This is not an unusual finding as genetic mouse models often only partially recapitulate the pathologies associated to the human disease, even when the same genetic mutations are reproduced in their genome by gene targeting (Cheon and Orsulic, 2011). Intrinsic physiological differences between the two species might account for the limitations in reproducing human phenotypes in mice. Nevertheless, the early symptoms and the premalignant condition of the human disease are fully reproduced in our model facilitating the design and evaluation of therapeutic strategies for its prevention.

Our model resembles other mouse models where hypoacidity and hypergastrinemic conditions have been shown to result in hyperplasia, and often dysplasia, of the gastric corpus, without the development of neoplasms in this region of the stomach (Fossmark et al., 2008). Previously described knockout (KO) mouse models have suggested that gastrin, and not achlorhydria, is responsible for these phenotypes (Franic et al., 2001; Judd et al., 2005; Zavros et al., 2005). However, in our model, lack of acid production, rather than gastrin deregulation, is more likely to be the major cause of the observed alterations in the stomach. Our KI/KI mice developed hypergastrinemia secondary to reduced acid secretion (Fig. 3F). Moreover, serum gastrin concentration in the evening was still higher in KI/KI mice than that observed in WT mice in the morning (after overnight feeding). This suggests that there is loss of the normal feeding-related physiological gastrin response in KI/KI mice.

Acidification of the stomach by treatment with HCl water not only prevented the development of the phenotype, but also partially reverted it when administered to adult animals. This occurred despite a twofold persistent hypergastrinemia in treated KI/KI mice compared with WT mice, suggesting that low stomach pH is the primary cause of the phenotype observed.

Therefore, our model supports the notion that the relationship between achlorhydria and hypergastrinemia and how they affect stomach hyperplasia and tumor development is still not fully understood.

### Comparison of human and mouse phenotypes

KI/KI mice developed most of the analytical, clinical and premalignant characteristics that were previously observed in humans who harbored this mutation (ATP4a R703C; described in Calvete et al., 2015). In humans with classic sporadic type I gastric NETs, the hypergastrinemia results in the development of multifocal malignancies and progressive PC destruction. As a consequence, these individuals develop megaloblastic anemia resulting from vitamin B12 deficiency caused by the lack of IF production by PC cells. In contrast, IF was produced both in the human individuals who had atypical familial gastric NETs (Calvete et al., 2015), and in our mouse model. Moreover, iron-deficiency anemia instead of megaloblastic anemia was observed in both mice and humans, as a result of the poor iron absorption at lower stomach pH.

Although the mice did not develop gNETs, our result provides functional evidence that this mutation is responsible for the observed phenotype in both human and mouse through development of a gNET and the pre-neoplasic stage, respectively. We do not know if other modifier factors are required in addition to the *ATP4a* mutation to induce the development of NETs in the KI/KI mice. However, *Helicobacter pylori* infection or gastric inflammation as non-genetic factors were ruled out in humans, and other genetic factors such as mutations in *REG1α*, *MEN1* or *SMAD4* – genes involved in other gastric or NE cancers – were not mutated in human individuals. Other studies will be necessary in order to clarify the involvement of these genetic and non-genetic factors.

In addition, we suggest that the differences between human and mouse in response to hypergastrinemia could be an important factor for the development of gNETs as an overexpression of SSTR2 signal was observed in human tumors (Calvete et al., 2015), whereas in the mouse model SSTR2 was not observed to be overexpressed in individual ECL cells. Instead, the increased ratio of SST-^68^Ga absorbed in the stomach to total SST-^68^Ga absorbed suggests that there is an overall increase in the number of ECL cells in the mutant mice in response to achlorhydria and hypergastrinemia, rather than an increase in the number of SSTR2 receptors per cell. This is consistent with the gastric mucosa hyperplasia (higher total cell number) observed in KI/KI mice. However, these differences must be deeply explored.

### Gland architecture

No hyperplasia or increased expression of gastrin or Ki67 were observed in the gastric antrum of KI/KI mice, although gastrin concentration was not observed to decrease during the non-feeding period in KI/KI mice (Fig. 3F), therefore suggesting that hypergastrinemia results from a more persistent and less feeding-dependent gastrin production by G-cells rather than from an increase in the G-cell population in this model.

In the gastric corpus of KI/KI mice, PC viability and number were not compromised in contrast to what has been previously described in *Atp4a* KO mice (Spicer et al., 2000) and in the hypochlorhydric and gastrin-deficient mice described by Zavros et al. (2005). Differences observed between the *Atp4a* KO mouse and our KI model might be related to the absence or non-functionality of the ATP4a protein, respectively. In the KO mice, the protein is not produced and this might affect PC viability in a different way than the point mutation here described.

PCs have been described as regulating the biology of the niche for cells of the gastric oxyntic glands and the maturation and differentiation of epithelial neck (isthmus) cells to chief cells (base of mucosa) (Bredemeyer et al., 2009). Disruption of this lineage patterning has been correlated with the development of stomach cancer. Achlorhydria in KI/KI mice altered the architecture of the oxyntic glands; moreover, cell proliferation patterns and cell localization were also altered in KI/KI mice and a delocalized IHC signal of Ki67 has been described in somatostatin-deficient mouse models (Zavros et al., 2005). This delocalization suggests that progenitor cells (neck mucosa cells) do not differentiate adequately throughout the gland. Therefore, achlorhydria seems to induce cell delocalization along the oxyntic gland and glandular metaplasia.

### Treatment and preclinical applications

In KI/KI mice, the achlorhydria that resulted from the missense mutation in the proton pump was partially corrected by administering acidified water in both prevention and reversion scenarios. The dysplasia, cellular delocalization and glandular metaplasia observed in the stomachs of untreated KI/KI mice disappeared after treatment with acidified water (Fig. 4B,E,F, Fig. 5B,E,F). Hypergastrinemia and iron-deficiency anemia, which result from gastric achlorhydria, were also not as pronounced in treated compared with non-treated KI/KI mice. Therefore, achlorhydria seems to cause the observed phenotype. We also partially restored the iron-deficiency anemia in treated KI/KI mice.

Treatment for gastric NETs can be expensive and the aggressive forms require major surgery that diminishes life quality. Our results indicate that the phenotype of KI/KI mice can be reverted, at least to some extent, by normalization of the gastric pH. We speculate that by administering a treatment that induces a more acidic gastric pH without damaging other tissues, the development of gastric pathology in humans might be prevented or reverted effectively and at low cost. Our findings might also open a new window for a cheap and non-aggressive alternative to reduce the impact of gastric pathologies and other pathologies that arise from achlorhydria or hypergastrinemia. Clinical application of this finding for treatment of gastric pathologies is therefore immediately inferred, but further studies are required for establishing the best treatment for individuals with gastric NETs.

## MATERIALS AND METHODS

### Study approval

All animal procedures were previously approved by CNIO (Register number: IACUC.043-2014) and the Institutional Animal Ethics Committee (Register number: PROEX292/14).

### Knockin mouse construction

Mice were housed in a specific pathogen-free (SPF) barrier facility. C57Bl/6JOlaHsd mice were obtained from the in-house colony. The p.R702C mutation in the *Atp4a* gene was introduced into the mouse germ line by gene targeting in the G4 ESC line (George et al., 2007) following protocols previously described (Nagy et al., 2003). The targeting vector was designed to target exon 14 of the *Atp4a* gene, introducing the mutation [CGA (R) to TGC (C)] at position 702 and a silent mutation in codon 709 [CTG (L) to CTT (L)] to create a *Hind*III restriction site (AAGCTT) for easy genotyping. For clone selection, a Frt-flanked PGK/bg2-neo^R^ cassette was introduced into the intron preceding 5′ to exon 13, with a transcriptional orientation opposite to that of the *Atp4a* gene. Homology arms were obtained from BAC RP23-72M20 and the lengths were 4.8 kb and 6.0 kb for the 5′ and 3′ arms, respectively (Fig. S1). Clones selected in G418 (200 µg/ml) for one week were screened by Southern blot using external probes amplified from the BAC DNA following the strategy described in Fig. S1. The 5′ external probe (289 bp) was amplified using primers forward 5′-CCCTGTCTCCAAAAACCAAA-3′ and reverse 5′-CGGCTTTGTGCATAGGAGTT-3′. The 3′ external probe (366 bp) was amplified using primers forward 5′-AAAACACACCCCCATTTGAA-3′ and reverse 5′-CTTTTGCCTTCCTCAGATGC-3′. Of 147 clones screened, four were positive for recombination in both arms and three of these were positive for both mutations in exon 14. Germ line-transmitting chimeras were generated from two of the mutant clones that contained the p.R702C mutation by morula aggregation. The knockin line was established from chimeras derived from one of these clones. To delete the PGK/bg2-neo^R^ cassette, heterozygous mice (KI/+) were crossed with Tg.CAG-Flp females (Nagy et al., 2003) and the Flp-mediated excision of the PGK/bg2-neo^R^ cassette was verified by Southern blot (Rodríguez et al., 2000). After cassette excision, the line was maintained by crossing heterozygous (KI/+) mice. Littermates of the three genotypes, WT, KI/+ and KI/KI, were used in the experiments. The line was maintained in a 129S6/SvEv, C57BL/6J mixed background.

Mouse genotyping was performed by PCR. Primers were designed to amplify sequences flanking the Frt site left in intron 12 after Flp-mediated recombination. Forward 5′-CCCTCCCTCAGCTTATTTCC-3′ and reverse 5′-GTCTCACCAGTCGCTGACAA-3′ primers amplified 166 bp and 233 bp fragments from WT and KI alleles, respectively (Fig. 1F). Genotyping was performed from DNA extracted from tail tissue (DNeasy Blood and Tissue Kit, Qiagen, 69504) at weaning.

### Blood samples

Blood was collected from mice by retro-orbital bleeding between 9:00 and 10:00 am to reduce feeding-related differences. Plasma and serum were obtained from blood after 10 min of centrifugation at 1077 ***g*** in an Eppendorf centrifuge at room temperature and were collected in 2 ml heparin-tubes (BD Vacutainer, 368494) and Eppendorf tubes, respectively.

### Macroscopic analysis

At necropsy, stomachs were carefully dissected, measured and weighed after elimination of food contents. Stomach pH was measured using a colorimetric pH indicator (0-14) strip. After dissection, a pH strip was introduced into the stomach before rinsing the tissue.

### Echography

Four different regions were assessed: corpus, antrum, pylorus and duodenum junctions. During the ultrasound study, mice received general anesthesia (isoflurane 2-3%). A Vevo 770 ultrasound imaging platform (Visualsonics) and a RMV 703 transducer were used.

### Gastrin measurement

Serum samples were assayed for total amidated gastrin concentrations by radioimmunoassay using antibody L2 (which reacts with G-17 and G-34 but not with progastrin or Gly-gastrins) and I-G-17 as previously described (Dockray et al., 1991). The upper limit of the reference range for serum gastrin in normal mice is 150 pM in this assay.

### Ferritin measurement

Ferritin was measured in plasma by using a hematology analyzer (ABX Pentra 400 from Horiba Medicals). For ferritin measurement, we used ABX Pentra Ferritin 2 CP (A11A01900), which uses ABX Pentra Immuno II Control L/H as control (A11A01622) and is calibrated with ABX Pentra Ferritin Calibrator (A11A01619).

### PET-CT imaging and biodistribution

The peptide DOTA-D-Phe1,Tyr3-octreotate (DOTA-TATE), a somatostatin analog, which has a high affinity for the SSTR2 subtype somatostatin receptor, was labeled with ^68^Ga obtained from a ^68^Ge/^68^Ga radionuclide generator as previously described (Romero et al., 2016).

PET studies were performed in an Argus PET-CT (SEDECAL). Images were acquired for 30 min under anesthesia (2-2.5% isoflurane in 100% O_2_ at a flow rate of 5% using a Fluovac device) 60 min after intravenous injection of ^68^Ga-DOTA-TATE (0.2-2 MBq). PET images were corrected for random events and scatter without attenuation correction, reconstructed using the 2D-OSEM algorithm and fused with the corresponding CT images.

For biodistribution studies, organs were excised, wet-weighed and counted for radioactivity with a gamma-counter (2470 Wizard2, PerkinElmer), along with a standard sample of the injected dose. Tissue activity was expressed as percentage of the injected dose per gram of tissue (%ID/g).

### Histology and immunohistochemistry

Stomachs and esophagi were collected, fixed in 10% formalin and embedded in paraffin. Blocks were cut into 5-μm thick sections. Details of the antibodies, incubation conditions and antigen retrieval methods are given in Table S1.

### Treatment with acidified water

Artificial acidification of the gastric juice was achieved by providing acidified water (pH: 2.6-2.8) to the mice as drinking water for the times established. Water was acidified by adding 0.215 ml of pure HCl (12N) per liter of water (pH 7) after autoclaving.

### Statistical analysis

Significance of differences among individuals grouped according to genotype (WT versus KI/KI) was evaluated with the non-parametric Kolmogorov–Smirnov test to determine normal distribution of values within the groups. Student's *t*-test was used for comparison of normally distributed values among genotypes. Differences were considered to be significant when the exact *P*-value was <0.05.
